# Association between game metrics in a simulation game and nursing students’ surgical nursing knowledge – a quasi-experimental study

**DOI:** 10.1186/s12912-023-01668-0

**Published:** 2024-01-02

**Authors:** Jaana-Maija Koivisto, Tuija Buure, Janne Engblom, Kristiina Rosqvist, Elina Haavisto

**Affiliations:** 1 Faculty of Medicine, University of Helsinki, PO BOX 20, Tukholmankatu 8B, 00014 Helsinki, Finland; 2 Metropolia University of Applied Sciences, Myllypurontie 1, 00920 Helsinki PL 4000, 00079 Metropolia, Helsinki, Finland; 3 Turku School of Economics, Department of Mathematics and Statistics, University of Turku, 20014 Turku, Finland; 4 Department of Health Sciences, Tampere University, Arvo Ylpönkatu 34, 33520 Tampere, Finland

**Keywords:** Surgical nursing, Knowledge, Simulation game, Virtual simulation, Game metrics, Nursing students, Nursing education, Quasi-experimental study

## Abstract

**Background:**

Simulation games are effective for acquiring surgical nursing knowledge during education by offering possibilities to learn theoretical knowledge through practical patient scenarios, thus preparing students for demanding surgical nursing care. Game metrics stored in the game system enable assessment of students’ behaviour while gameplaying. Combining game metrics with the assessment of a student’s surgical nursing knowledge allows versatile information to be obtained about the student’s learning outcomes. However, studies on game metrics stored in systems and their relationship with learning outcomes are scarce.

**Methods:**

The aim here was to evaluate the association between game metrics in a simulation game and nursing students’ surgical nursing knowledge. Nursing students from three universities of applied sciences in Finland participated in a one-week simulation gameplaying intervention that included five surgical nursing scenarios. Students’ surgical nursing knowledge was investigated with a quasi-experimental, one-group, pre- and post-test design using a surgical nursing knowledge test. In total, 280 students filled in the knowledge tests. In addition, cross-sectional game data were collected at a single time point between pre- and post-tests. The data were analysed with descriptive statistics and multivariate analysis methods.

**Results:**

Students’ surgical nursing knowledge improved with the intervention. The total number of playthroughs was 3562. The mean maximum score was 126.2 (maximum score range 76–195). The mean playing time of all playthroughs by all players was 4.3 minutes (*SD* = 81.61). A statistically significant association was found between mean score and knowledge test total score (*p* < 0.0072), but no significant association emerged between mean playing time and knowledge test total score.

**Conclusion:**

The results indicated that the higher the mean score the better the students’ surgical nursing knowledge in the knowledge test. This study did not show that the time spent playing had an impact on students’ post-playing knowledge. Our findings support the idea that game metrics can be used in performance evaluation and the results can be used to improve nursing students’ readiness for challenging preoperative and postoperative clinical situations.

## Introduction

Surgical nursing care is demanding since the patient’s situation may change suddenly, especially in the postoperative phase, and nurses must make complex decisions in time-constrained situations [1, 2]. Cognitive skills needed in surgical nurses include early recognition of physiological deterioration, confirming physiological deterioration, initiating rescue, securing medical assistance, and rescuing the patient [1]. Especially among newly graduated nurses, the early recognition of physical deterioration is difficult due to limited exposure to patients with complex problems [1]. Therefore, students must gain experience in coping with challenging situations in clinical practice already during their training. With the current global shortage of nurses [3], it is even more important to create conditions for the successful learning experience in clinical practice so that students remain in the nursing field [4].

To prepare nursing students for challenging clinical situations, online interactive software tools that combine theoretical pre- and postoperative nursing care content and practical skills are needed [5–8]. In recent years, teaching and learning methods for surgical nursing have diversified. Recent scoping review revealed that teaching pre- and postoperative content has included human patient simulations, virtual simulations, web-based learning, and case studies [5]. Interactive and motivating virtual simulations may benefit students in their clinical preparedness since they promote systematic patient assessment [9] which is important skill for surgical nursing. In addition, a desktop virtual reality application for learning preoperative handover enables undergraduate nursing students to be active in the learning activity, which facilitates perceived learning outcomes [10].

In addition to the benefits related to the learning process, it has been shown that simulation games are effective for acquiring surgical nursing knowledge during education (Anonymised, accepted for publication). Previously, we conducted a quasi-experimental study, including a simulation game group (*n* = 140) and a control group (*n* = 136) and pre- and post-test measurement of the level of surgical nursing knowledge with a surgical nursing knowledge (SNK) test (Anonymised, accepted for publication). The results revealed that both groups had better surgical nursing knowledge after the intervention than before the intervention, but the change was greater in the simulation game group than in the control group.

One benefit of using simulation games in education and research is related to the information they store about students’ behaviour while playing and the information it produces to evaluate learning outcomes. This information, so-called game metrics, can be used in performance evaluation in education. Game metrics measure all actions taken by a player during playing, for example, the player’s location in the game environment and interactions with other game characters [11]. In educational research, game metrics have mostly focused on playing time [11–13], the number of games played, the number of completed tasks, and points [12–14].

The educational value of game metrics lies in their providing information to students and teachers about students’ skills and knowledge [14, 15] and students’ learning and competency [16]. Game metrics could also be used to determine the optimal playing time to achieve good performance. In a learning context other than nursing education, one study demonstrated that the quality of the learning outcome was positively correlated with the time spent and the number of objects placed during gaming [13] but by contrast, another study found no correlation between time spent on execution and learning effectiveness [17]. In nursing education, it has been found that nursing students’ mean scores in a nursing simulation game were significantly associated with clinical reasoning skills [14]. Further, it has been reported that total time spent by students on the simulation game correlated with learning [18] and the more time students spent on playing a scenario the better their mean score was [15]. This implies that students achieve higher scores when engaging for a longer time in the scenarios, thus reflecting better performance.

However, although virtual simulations and games are increasingly used in nursing education, published research on the game metrics stored in the systems and their connection to learning outcomes is lacking. The experimental work presented here is one of the first studies to fill this knowledge gap.

## Methods

### Aim

The aim was to evaluate the association between game metrics in a simulation game and nursing students’ surgical nursing knowledge.

### Research design

Investigating nursing students’ surgical nursing knowledge comprised a quasi-experimental, one-group, and pre- and post-test design [19]. Cross-sectional game data were collected at a single time point between the pre- and post-tests. The combination of these research designs adds value to the much-used within-subjects design in educational research in the field of nursing [20].

### Setting and participants

Nursing students participating in surgical nursing courses between 1 March 2018 and 31 May 2019 were recruited using purposive sampling [19] from three universities of applied sciences (UAS) in Finland. The total population of students in the surgical nursing courses in the three UAS was 800. The goal of the surgical nursing course in the Finnish curriculum is for students to master basic knowledge and skills in different phases of preoperative and postoperative care of patients requiring surgical intervention (surgery and surgical specialties). In addition, during the course the students develop their abilities to respond to individual needs of surgical patients by planning, implementing, and assessing appropriate nursing care. At the time of data collection, 400 students played a simulation game as a part of the course’s learning activities, and these students were requested to participate’ in the study. Criteria for selecting the students were as follows: students were either first- or second-year students, they participated in a surgical nursing course, they played a simulation game including five pre- and postoperative nursing scenarios, and they voluntarily participated in the study. Recruitment was carried out by researchers and contact teachers at the beginning of each course. The data collection period was 1.5 years.

### Intervention

**Fig. 1:**
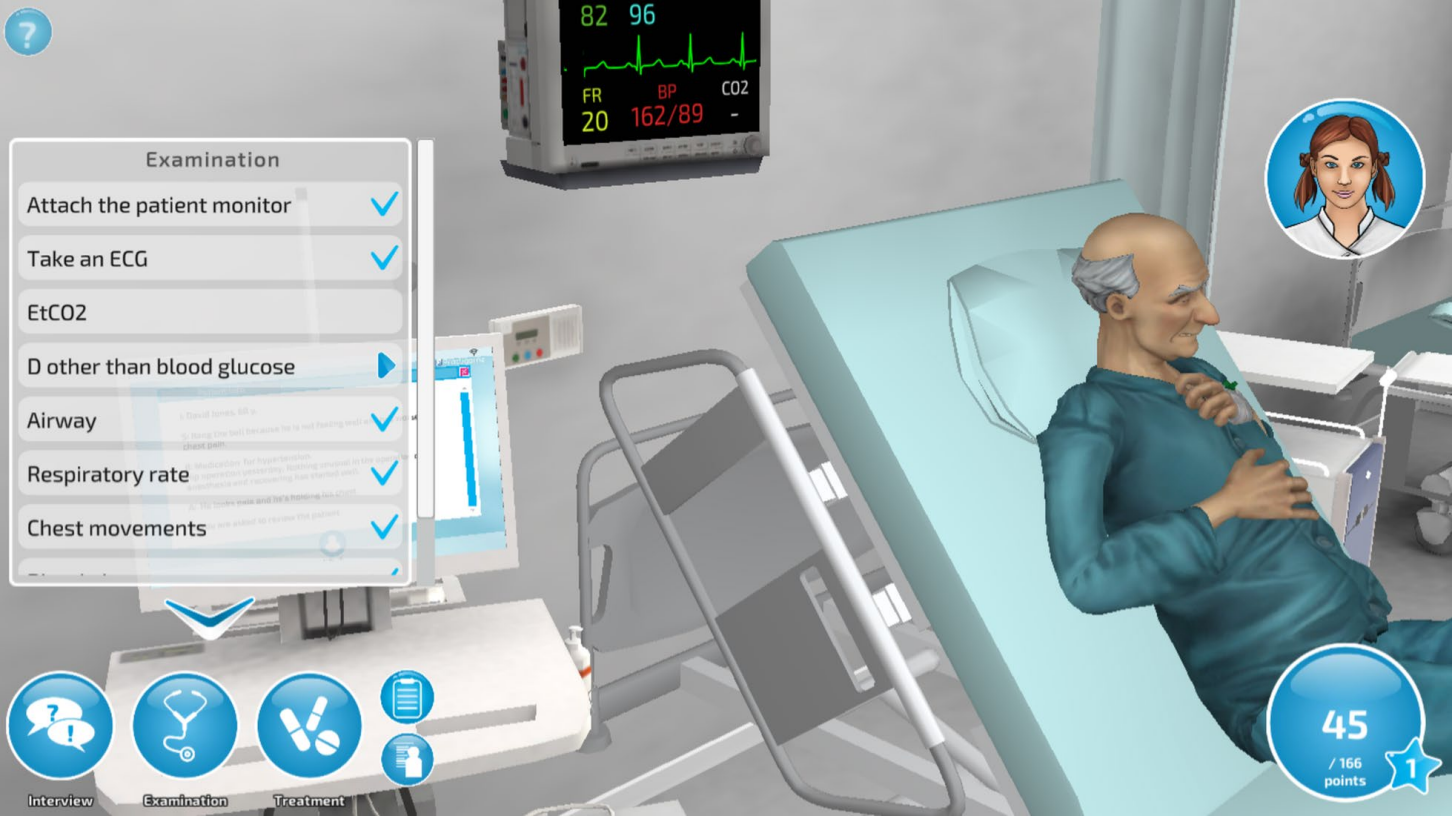
Screenshot of the simulation game (English version)

The intervention involved playing a simulation game for a week. The intervention week started with filling in an electronic questionnaire, including a surgical nursing knowledge (SNK) test (pretest) and background information. The game was a desktop virtual simulation (Fig. 1), including game elements that have been found to promote learning in the nursing context [21, 22]. The simulation was developed with a game engine that enables the visualization of an authentic nursing environment with graphics of realistic equipment and animations indicating a patient’s reactions, gestures, and facial expressions [23]. An evidence-based content was created for the five surgical nursing scenarios corresponding to curriculum objectives of pre- and postoperative patient care (Anonymised, accepted for publication) (Table 1). Action points in the game included interviewing the patient, assessing the patient’s clinical condition, and implementing nursing interventions by interacting with the game via clicking a computer mouse from the three main menus of interview, examination, and treatment. Students received a score for each choice that they made during their scenario performance, earning points for correct actions and losing points for incorrect actions [15]. Students were able to repeat scenarios according to their own motivation and learning goals. At the end of the intervention week, the participants filled in the SNK test again (post-test).

**Table 1:** Learning goals and contents of the surgical patient scenarios in the simulation game

**SURGICAL PATIENT SCENARIOS IN THE SIMULATION GAME**				
**LEARNING GOALS**				
Assess the patient’s preoperative state using the ABCDE approach.				
Identify the special characteristics of the care of the emergency patient.				
Identify signs of infection.				
Prepare patient for surgery.				
Assess the patient’s postoperative state using the ABCDE approach.				
Recognize the symptoms of hypovolemia.				
Recognize postoperative complications.				
Assess the patient’s pain.				
Identify the NEWS criteria.				
Implement pain management.				
Implement nursing interventions.				
Perform blood transfusion in a patient-safe manner.				
Reconstitute the blood product for the patient.				
Monitor the patient’s well-being during transfusion.				
Identify symptoms of anaphylaxis.				
**SCENARIO 1**	**SCENARIO 2**	**SCENARIO 3**	**SCENARIO 4**	**SCENARIO 5**
Preoperative assessment of an orthopaedic surgery patient	Postoperative haemorrhage and hypovolemia	Postoperative assessment peripheral artery bypass surgery patient	Identification of disorders of vital signs according to NEWS in postoperative nursing	Blood transfusion of surgical patient
Max score 115	Max score 95	Max score 195	Max score 150	Max score 76
A 70-year-old man with osteoarthritis in the left knee, type 2 diabetes mellitus, and previous heavy alcohol use. The patient has come to the surgical ward for the interview and preparations for the day of surgery.	A 70-year-old man with hypertension. Intestinal resection was performed under general anaesthesia today. The patient has been in the ward for 2 hours. The patient has IV infusion, catheter, and epidural pain medication. There is no discharge from the wound dressings.	A 70-year-old man with hypertension, Type 2 diabetes mellitus and ASO. Bypass surgery on the lower limb was performed yesterday.	A 70-year-old man. Laparoscopic removal of the gallbladder was performed under general anaesthesia today. The patient has a catheter and there is no discharge from the wound dressings. The patient rings the alarm bell.	A 70-year-old man with hypertension and hypercholesterolemia. Transurethral Resection of the Prostate (TURP) was performed on the previous day. Today, the patient has low haemoglobin (84 g/l). The doctor has ordered a blood transfusion. The patient is tired and has pain in the wound area.

### Data collection

**Fig. 2:**
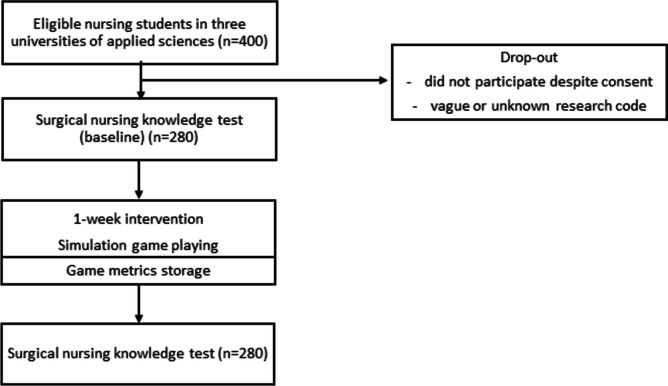
Flowchart of quasi-experimental research design

Data on educational background were collected as background education since in Finland, eligible applicants for degree programmes in a university of applied sciences have completed the general upper secondary curriculum or passed the examination referred to in the Finnish Matriculation Examination, have been awarded an initial vocational qualification, further vocational qualification or specialist vocational qualification or have completed studies abroad which give eligibility for higher education in the country in question [24]. In addition, since some of the nursing students may have had previous work experience as a practical nurse (vocational qualification) work experience in social and health services were collected as background information. A knowledge test was used in the surgical nursing course to assess students’ knowledge about the issues covered in the course. The level of surgical nursing knowledge was assessed by pre- and post-tests with the SNK test (Anonymised, accepted for publication). Altogether 280 nursing students filled in the SNK test (Fig. 2). The SNK test consisted of six subscales and 60 items (Table 2). The items were rated on a dichotomic scale (correct or incorrect). The maximum score was 60.

**Table 2:** Nursing students` surgical nursing knowledge (*N* = 280)

Surgical Nursing Knowledge test score	M	SD	M	SD
SNK total	43.01	3.82	44.33	3.18
SNK 1 Blood transfusion of surgical patient	7.16	1.18	6.44	1.08
SNK 2 Postoperative haemorrhage and hypovolemia	7.10	1.40	7.85	1.01
SNK3 Preoperative assessment of an orthopaedic surgery patient	8.04	1.09	8.60	0.99
SNK4 Postoperative assessment peripheral artery bypass surgery patient	6.22	1.27	7.38	1.27
SNK5 Pain management of surgical patient	6.38	1.41	6.88	1.49
SNK6 Identification of disorders of vital signs according to NEWS in postoperative nursing	8.11	1.22	7.18	1.21

Students’ performance in the simulation game was evaluated with game metrics stored in the simulation game database. The game metrics included the following quantitative measures: the mean score and the mean playing time of all playthroughs by all students [14, 15]. Playthroughs refer here to all scenarios played by all students, whether or not the scenario was played from start to finish.

### Data analysis

Descriptive statistics were used to present the characteristics of the study participants. Associations between the mean score and mean playing time and the SNK test (post-test) were examined by using a linear regression model adjusted by educational background, work experience in social and health services, work experience in social and health services, and surgical nursing knowledge (pre-test). A statistical analysis was performed using SAS version 9.4.

## Results

In this study, a slight majority of students (58.6%) had completed the Finnish Matriculation Examination. More than half of the students (60%) had less than 1 year of work experience in social and health services.

### Nursing students’ surgical nursing knowledge

The whole group had better total surgical nursing knowledge after the intervention than before the intervention (*M* = 44.33, *SD* = 3.18). Before the intervention, the greatest knowledge was in sub-areas SNK 6 ‘Identification of disorders of vital signs according to NEWS in postoperative nursing’ (*M* = 8.11, *SD* = 1.22) and SNK 3 ‘Preoperative assessment of an orthopaedic surgery patient’ (*M* = 8.04, *SD* = 1.09). The knowledge was poorest in sub-areas SNK 4 ‘Postoperative assessment of a peripheral artery bypass surgery patient’ (*M* = 6.22, *SD* = 1.27) and SNK 5 ‘Pain management of a surgical patient’ (*M* = 6.38, *SD* = 1.41). After the intervention, the greatest knowledge was in the sub-area SNK 3 ‘Preoperative assessment of an orthopaedic surgery patient’ (*M* = 8.60, *SD* = 0.99), whereas the poorest knowledge was in the sub-area SNK 1 ‘Blood transfusion of a surgical patient’ (*M* = 6.44, *SD* = 1.08) (Table 2).

### Association between mean score and mean playing time and surgical nursing knowledge

The total number of playthroughs was 3562. The mean score of all playthroughs by all players was 95.27 (*SD* = 14.59). The mean maximum score was 126.2 (maximum score range 76–195). The mean playing time of all playthroughs by all players was 4.3 minutes (*SD* = 81.61). A statistically significant association was found between mean score and knowledge test total score (*p* < 0.0072) (Table 3). Regarding knowledge test sub-areas, a statistically significant association was found between mean score and SNK 5 ‘Pain management of a surgical patient’ (*p* < 0.0030**)** but not in other sub-areas. No statistically significant association was found between mean playing time and knowledge test total score.

**Table 3:** Association between the mean score and mean playing time and the surgical nursing knowledge

	**SNK total**			**SNK 1** Blood transfusion of surgical patient			**SNK 2** Postoperative haemorrhage and hypovolemia		
	Estimate^a^	s.e.	p.^b^	Estimate^a^	s.e.	p. ^b^	Estimate^a^	s.e.	p. ^b^
Mean score	0.03364	0.01241	**0.0072**	0.002064	0.004550	0.6505	0.005360	0.004244	0.2077
Mean playing time	0.002033	0.002134	0.3415	−0.00004	0.000809	0.9580	0.000128	0.000741	0.8635
	**SNK 3** Preoperative assessment of an orthopaedic surgery patient			**SNK 4** Postoperative assessment peripheral artery bypass surgery patient			**SNK 5** Pain management of surgical patient		
	Estimate^a^	s.e.	p. ^b^	Estimate^a^	s.e.	p. ^b^	Estimate^a^	s.e.	p. ^b^
Mean score	0.001951	0.004165	0.6398	0.006479	0.005449	0.2355	0.01676	0.005600	**0.0030**
Mean playing time	0.000843	0.000729	0.2490	−0.00048	0.000964	0.6188	0.001393	0.000993	0.1619
	**SNK 6** Identification of disorders of vital signs according to NEWS in postoperative nursing								
	Estimate^a^	s.e.	p. ^b^						
Mean score	0.007711	0.005101	0.1318						
Mean playing time	0.000357	0.000895	0.6904						

^a^Estimates are regression slopes. Estimates are adjusted by educational background, work experience in social and health services and surgical nursing knowledge (pre-test)

^b^*p* < .05 was considered significant

## Discussion

This study was undertaken to evaluate the association between game metrics in a simulation game and nursing students’ surgical nursing knowledge. These experiments confirmed that surgical nursing knowledge of nursing students was better after the intervention than before the intervention, as expected, also supporting our previous findings (Anonymised, accepted for publication). Using a knowledge test to measure surgical knowledge objectively demonstrates an increase in knowledge, although it must be noted that educational interventions generally have the desired effect on learning outcomes. In this study, the association between background information and game scores and knowledge test was not investigated but work experience in the social and health services that some of the students had may have had a slight influence on their scores in the game and knowledge test. Although it is unlikely, it is worth investigating more in the future.

Nevertheless, these results add to the rapidly expanding data for the educational value of virtual simulations [5, 7] in learning key competence areas of nursing such as surgical nursing. The contents of the simulation game scenarios corresponded to the cognitive skills required from surgical nurses [1], and thus, it could be cautiously stated that the simulation game supported nursing student knowledge in these cognitive areas. The scenarios also enabled students to practice a certain systematic framework or protocol, as in other Nordic studies [10, 25], and repeating the same content in a virtual simulation has been found to promote the learning of nursing students [9, 26].

As in many earlier reports [11–13], the game metrics in this study focused on scores and time spent playing. Regarding mean score and learning outcomes, the results corroborate the findings of a previous study reporting that mean scores were significantly associated with learning outcomes, in that case clinical reasoning skills [14]. This study showed that by playing a simulation game it is also possible to acquire knowledge and therefore, in addition to traditional ways of acquiring knowledge, such as lectures and written assignments, simulation games can be used in nursing education to strengthen student knowledge base on surgical nursing. This, in turn, can contribute to successful learning experiences in clinical practice which can potentially engage students in the nursing field [4].

Regarding mean playing time, these results did not confirm the association between learning outcome and time spent playing, unlike an earlier study [13], but did confirm the findings of where no correlation was noted between time spent playing and learning effectiveness [17]. On the other hand, in our earlier study (Anonymised, accepted for publication), where we investigated the association between time spent playing and the mean scores achieved by students in different scenarios, the result showed that the more time students spent playing a scenario the better their mean score. Taken together, research results are inconsistent and warrant further investigations before conclusively stating that time spent playing is associated with positive learning outcomes. However, shorter playing time may also reflect better initial knowledge, i.e. superior knowledge at the outset will speed up the playing required to learn something new. Thus, more research is needed to determine the optimal playing time to achieve good performance.

A strength of the study is that two objective measures were used: a knowledge test and game metrics. Both have been used in previous studies [15] (Anonymised, accepted for publication). This avoided the bias that can arise from self-evaluation. However, these results may be biased due to the purposive sampling and the study being limited to three UAS in one country. In assessing the validity of the game metrics, it is essential to pay attention to data collection and interpretation of the results. The correct information must be collected about the player’s behaviour in the game. A reliable interpretation of the results requires a clear definition of the investigated objects, i.e. an explanation of what is to be shown with the measured objects. In this study, we determined in advance that information on the mean scores and mean playing time would be collected so that their connection to scores on the knowledge test could be examined. By combining game metrics with knowledge test data, the validity of the research result could be improved. However, one limitation of the study was the one-sided use of game metrics, with only mean playing times and mean scores being evaluated. A factor that weakens the validity of the findings is that some of the students played the scenarios for a very short time, reflected in the large standard deviation in playing times. These results, therefore, need to be interpreted with caution, and in the future game metrics should be used in a more versatile manner in the evaluation of learning outcomes.

## Conclusion

The main finding of this study was that the higher the mean score in the simulation game was the better the students’ surgical nursing knowledge was in the knowledge test. However, this study did not show that the time spent playing had an impact on students’ post-playing knowledge. The results show that it is possible to acquire surgical knowledge with playing the interactive simulation game. In addition, the results indicate that game metrics can be used to objectively evaluate performance in education. Taken together, these findings suggest that simulation games could be used to improve nursing students’ cognitive skills for challenging preoperative and postoperative clinical situations and game metrics could be used to evaluate learning outcomes.

## Data Availability

The quantitative datasets used and analysed in this study are available from the corresponding author on reasonable request.
